# Probing the Electron Transfer between iLOV Protein and Ag Nanoparticles

**DOI:** 10.3390/molecules25112544

**Published:** 2020-05-29

**Authors:** Xia Ran, Qianqian Zhang, Yu Zhang, Jin Chen, Zhongran Wei, Yulu He, Lijun Guo

**Affiliations:** 1 Institute of Micro/Nano Photonic Materials and Application, School of Physics and Electronics, Henan University, Kaifeng 475004, China; ranxia@henu.edu.cn (X.R.); zhangqianqian1992@126.com (Q.Z.); chenjin525525@126.com (J.C.); weizhongran@126.com (Z.W.); 2 Key Laboratory of Plant Stress Biology, Henan University, Kaifeng 475001, China; qikeli@hotmail.com

**Keywords:** Ag nanoparticles, iLOV protein, electron transfer, time-resolved fluorescence, tryptophan

## Abstract

Nanomaterials have been widely used in biomedical sciences; however, the mechanism of interaction between nanoparticles and biomolecules is still not fully understood. In the present study, we report the interaction mechanism between differently sized Ag nanoparticles and the improved light-oxygen-voltage (iLOV) protein. The steady-state and time-resolved fluorescence results demonstrated that the fluorescence intensity and lifetime of the iLOV protein decreased upon its adsorption onto Ag nanoparticles, and this decrease was dependent upon nanoparticle size. Further, we showed that the decrease of fluorescence intensity and lifetime arose from electron transfer between iLOV and Ag nanoparticles. Moreover, through point mutation and controlled experimentation, we demonstrated for the first time that electron transfer between iLOV and Ag nanoparticles is mediated by the tryptophan residue in the iLOV protein. These results are of great importance in revealing the function of iLOV protein as it applies to biomolecular sensors, the field of nano-photonics, and the interaction mechanism between the protein and nanoparticles.

## 1. Introduction

Nanotechnology has been widely used to detect biological processes and reveal their potential applications in many biological fields, including those involving biosensors, drug delivery, gene therapy, and disease diagnosis and therapy [1,2,3,4,5,6,7,8]. The interactions between nanoparticles and biomolecules play important roles in the abovementioned processes. Specifically, when biological molecules are in close proximity to nanoparticles, FRET (fluorescence resonance energy transfer), SET (surface energy transfer), or electron transfer can take place upon excitation of the donor, biomolecule or nanoparticle [9,10]. FRET occurs only when the emission spectrum of the donor overlaps with the absorption spectrum of the acceptor and distance between the donor and acceptor is within the 1–10 nm range. In the FRET process, the transfer efficiency decreases with the 6th power of distance. SET is most often observed between the metallic surface of nanoparticles and organic molecules. SET does not require spectrum overlap, and happens when the distance between donor and acceptor is larger than 15 nm. The efficiency of SET depends on the 4th power of distance. Electron-transfer efficiency relates to many factors, such as redox potential, but has no critical demand for the distance between donor and acceptor [9,11]. FRET, SET, and electron transfer govern many important biochemical processes [12,13], and it is essential to study the fundamental mechanism of the interplay between the nanoparticles and interacting biomolecules. Due to the complexity of biological systems, the pathway and mechanism of interactions between nanoparticles and biomolecules are diverse and not fully understood. Due to their special surface plasmon resonance property, Ag nanoparticles have attracted considerable attention in recent years [14,15,16], and have demonstrated potential applications in wound healing and the treatment of ulcers [14,17]. Many “green” methods, which have advantages over conventional methods involving chemical agents associated with environmental toxicity, for the synthesis of silver nanoparticles and compositions are now available, facilitating their application in biomedical fields [18,19,20]. The antibacterial effect of Ag nanoparticles is well known, and the toxicity of Ag nanoparticles may cause unfavorable side effects in organisms such as algae, fish, mammalian cells in vitro, and so on [21]. These side effects may limit the usage of Ag nanoparticles in biology and biomedicine unless the relevant interaction mechanism between Ag nanoparticles and protein is well understood. Therefore, it is important to study the interaction of Ag nanoparticles with biological molecules and to predict avenues for the design and synthesis of novel nano–bio composites in biomedical material science [22,23].

Genetically encoded fluorescence proteins have been used in a wide range of applications, such as metal sensing, in vivo cell and tissue imaging, fluorescence labeling, detection of protein–protein interactions, and protein–polysaccharide conjugation [24,25,26]. The iLOV (improved light-oxygen-voltage) protein is a mutant flavin-based fluorescent protein that contains a LOV (light-oxygen-voltage)-sensing domain with a non-covalently bound flavin mononucleotide (FMN) as its chromophore, and is considered an alternative to the genetically encoded fluorescence protein family due to its improved photophysical properties, rapid maturation of fluorescence, and increased thermal stability [26]. Compared with other green fluorescent proteins, iLOV possesses improved photophysical properties, fluorescence stability, and thermal stability which facilitate its application as a fluorescent reporter [27]. Metal nanoparticles can interact with the residues of certain amino acids, resulting in energy transfer between metal nanoparticles and proteins [28].

The iLOV protein has been widely used as a photoreceptor and photosensor, but its photoresponse mechanism is still not fully understood. Biochemical methods have revealed that the photoreaction of iLOV is due to light-induced electron transfer, and tryptophan is the electron donor; however, more experimental evidence is needed to support this view. In the present study, we constructed iLOV-W83A protein by mutating the only tryptophan in iLOV to alanine and demonstrated there was no electron transfer between Ag nanoparticles and iLOV-W83A, which supported the aforementioned view.

In this work, we investigated the interaction between fluorescent proteins and metal nanoparticles using the iLOV protein and Ag nanoparticles as a model system. The fluorescence quenching of iLOV arises from electron transfer rather than energy transfer to Ag nanoparticles, and demonstrated a dependence on the size of Ag nanoparticles. Additionally, the observed electron-transfer pathway mediated by tryptophan clarified the photoresponse mechanism of iLOV.

## 2. Results and Discussion

### 2.1. Properties of iLOV and iLOV-W83A

**Figure 1:**
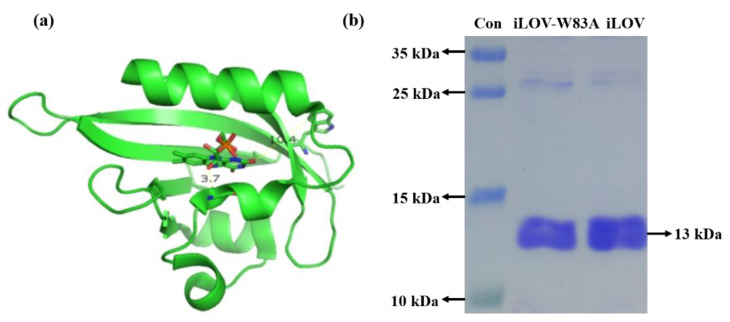
(**a**) Protein model of iLOV (improved light-oxygen-light); (**b**) SDS-PAGE analysis of the marker (Lane 1) and iLOV-W83A (with mutation of the only tryptophan in iLOV to alanine) (Lane 2) and iLOV (Lane 3) proteins.

The iLOV protein is an FMN-based protein with high stability. It consists of 113 amino acids and has a molecular weight of 13 kDa. iLOV-W83A has similar structures to iLOV, except that the sole tryptophan in iLOV was mutated to alanine in iLOV-W83A. Figure 1a shows the protein model of iLOV. The gel image of the iLOV and iLOV-W83A proteins is shown in Figure 1b. The absence of smearing in Figure 1b indicated the purity of the target protein. According to the migration of the iLOV and iLOV-W83A bands, the molecular weights of both iLOV and iLOV-W83A were approximately 13 kDa.

### 2.2. Properties of Ag Nanoparticles and Fluorescent Proteins

**Figure 2:**
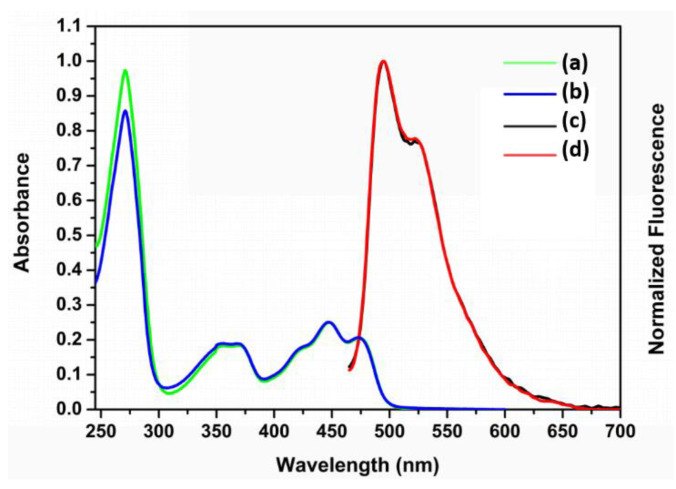
Absorption (**a**,**b**) and emission (**c**,**d**) spectra of iLOV and iLOV-W83A proteins.

The UV-vis absorption and fluorescence spectra of iLOV and iLOV-W83A proteins are shown in Figure 2. In the visible region, both iLOV and iLOV-W83A exhibited broad absorption bands with two maxima at about 447 and 470 nm with higher and lower intensity, respectively. The absorption band in the UVA region (peak at 350–370 nm) was attributed to the amino acids surrounding the flavin 7a-methyl group in the iLOV protein. The absorption in this region is rather sensitive to point mutations of the apoprotein [29]. The maximum emissions of both iLOV and iLOV-W83A localized at 495 nm with a well-pronounced shoulder at 532 nm, which originated from the oxidized FMN chromophore. The absorption characteristics of iLOV-W83A were almost the same as those of iLOV except for a minor difference at around 271 nm, which likely corresponded to the difference in absorption between tryptophan and alanine. These data strongly suggest that the only tryptophan residue in the iLOV amino-acid sequence was replaced by alanine without disturbing the protein structure.

**Figure 3:**
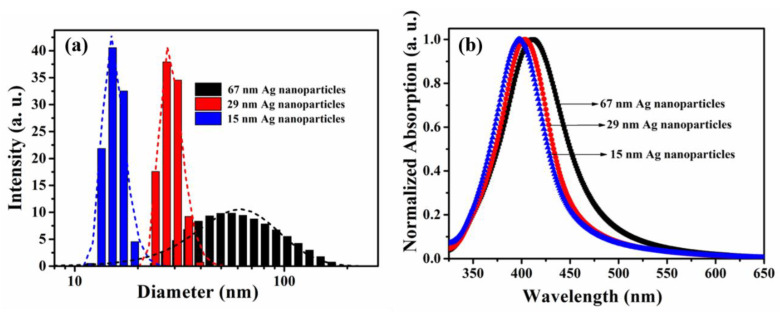
Normalized UV-vis absorption spectra (**a**) and size distribution (**b**) of Ag nanoparticles prepared at 0 °C, 35 °C, and 70 °C, respectively.

As shown in Figure 3a, the hydrodynamic particle size distribution of the three silver nanoparticles was monitored using the dynamic light scattering (DLS) method, demonstrating the mean hydrodynamic diameters of 67 nm, 29 nm, and 15 nm for the preparation temperatures of 0 °C, 35 °C, and 70 °C, respectively. The spectral absorption of Ag nanoparticles is largely attributed to surface plasmon resonance (SPR), and the SPR band is usually associated with the size, shape, and surface chemistry of the nanoparticles. According to the Mie theory, the absorption band position of spherical silver nanoparticles redshifts as particle size increases, and the bandwidths are correlated to the size distribution in the absorption spectrum [30]. As shown in Figure 3b, the three kinds of nanoparticles all displayed a strong SPR absorption band around 410 nm and, with increasing particle size, the absorption spectra showed a certain degree of redshift. This was in accordance with the aforementioned theoretical description.

### 2.3. Steady-State Fluorescence Spectroscopy of iLOV–Ag Nanoparticle Mixtures

**Figure 4:**
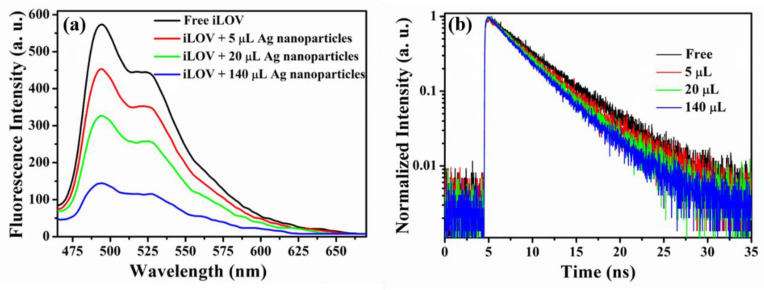
(**a**) The fluorescence emission spectra of iLOV with increasing Ag nanoparticle concentration. (**b**) Time-resolved fluorescence of iLOV with increasing Ag nanoparticle concentration.

The spectroscopic properties of fluorescent molecules, such as quantum yield and fluorescence intensity, change when they came close or adsorption to metal nanoparticles [31,32]. Figure 4a shows the fluorescence spectra of iLOV protein and iLOV–Ag nanoparticle mixtures with excitation at 430 nm. Fluorescence quenching of iLOV was observed in the presence of 29 nm Ag nanoparticles, and the quenching effect increased with the concentration of added Ag nanoparticles, while the spectral shape remained the same as that of free iLOV. This observation indicates there was energy transfer (FRET or SET) or electron transfer between iLOV and Ag nanoparticles.

### 2.4. Fluorescence Lifetime of iLOV and iLOV–Ag Nanoparticle Mixtures

To further investigate the interaction between iLOV and Ag nanoparticles, the fluorescence lifetimes of iLOV and iLOV–Ag nanoparticle mixtures were measured using time-correlated single-photon counting using variable amounts of 29 nm Ag nanoparticles (Figure 4b). Compared with the free iLOV protein, we observed that the fluorescence lifetime of iLOV-Ag nanoparticles was shortened with increasing Ag nanoparticle concentration, clearly demonstrating the interaction between iLOV and Ag nanoparticles.

To quantitatively characterize the transfer process, a biexponential decay equation was used to fit the experimental time-resolved fluorescence decays, while the fluorescence decay curve of iLOV protein was fitted with a monoexponential function.

Fitting the monoexponential fluorescence decay curve of iLOV without Ag nanoparticles resulted in a lifetime τ_1_ of 4.8 ns, which was close to previously reported results [33]. With the presence of Ag nanoparticles, the fluorescence of iLOV in mixture was expected to demonstrate a biexponential or multiexponential decay, arising from free iLOV protein and iLOV–Ag nanoparticles. Therefore, the fluorescence lifetime of iLOV adsorbed on Ag nanoparticles could be extracted from fitting the fluorescence dynamics of the donor iLOV. Assuming that the interaction is uniform, we biexponentially fitted the decay curve of iLOV. Besides the 4.8 ns component of free iLOV protein, the additional component in the lifetime arose from the interaction between iLOV and Ag nanoparticle, which was dependent on the concentration and size of Ag nanoparticles. The experimental results demonstrated that, when the concentration of iLOV was below 1 × 10^−5^ M, the fluorescence lifetime of free iLOV was independent of concentration, and a shorter lifetime due to electron transfer was obtained from fitting the fluorescence dynamics of iLOV–Ag nanoparticles. According to the fitting results, the fluorescence lifetime of iLOV protein adsorbed onto Ag nanoparticles was determined to be 2.7 ns. The fitting parameters of iLOV fluorescence dynamics with different amounts of 29 nm Ag nanoparticles are summarized in Table 1.

The reduction of radiative decay rate or the increase of nonradiative decay rate leads to the fluorescence quenching of the donor protein, and usually depends on the size of the nanoparticle and the distance between the nanoparticle and protein [34,35]. Fluorescence resonance transfer (FRET), surface energy transfer (SET), and electron transfer between Ag nanoparticles and iLOV are the three possible processes leading to the shortening of the iLOV fluorescence lifetime. FRET occurs only when there is an overlap between the emission spectra of the donor and the absorption spectra of the acceptor. However, according to Figure 2 and Figure 3a, there is no obvious overlap between the emission spectrum of iLOV and the absorption spectrum of 29 nm Ag nanoparticles. Therefore, the possibility that FRET leads to the fluorescence quenching of iLOV was preliminarily excluded, and SET or electron-transfer processes between silver nanoparticles and iLOV, which have no requirement for spectral overlap between donor and acceptor, were suggested as likely processes causing iLOV fluorescence quenching.

### 2.5. The Interaction of iLOV Protein with Different Sizes of Ag Nanoparticles

**Figure 5:**
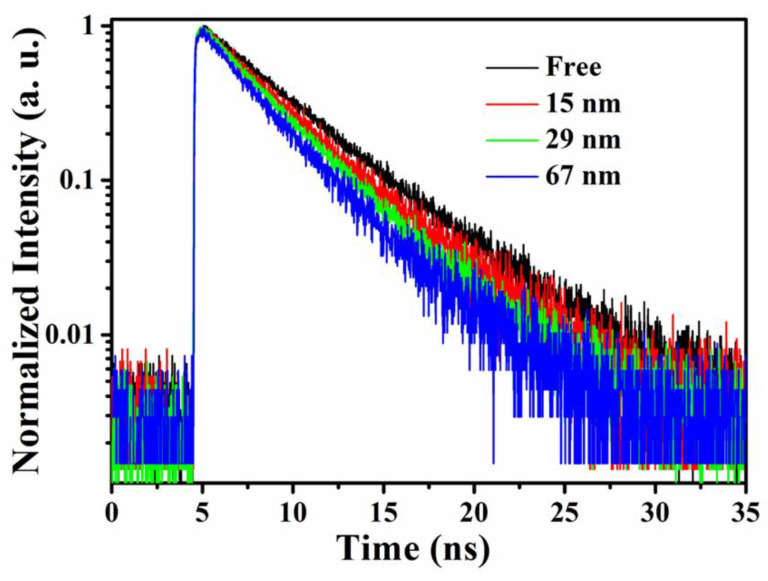
Time-resolved fluorescence of iLOV mixed with differently sized Ag nanoparticles (140 uL).

To gain more insight into the interaction process between iLOV and Ag nanoparticles, we investigated the fluorescence quenching of iLOV protein with the presence of differently sized Ag nanoparticles. The fluorescence decay time of 4.8 ns (τ_1_) for free iLOV protein was determined by fitting the data shown in Figure 5 with a single exponential function. However, the photoluminescence decay of iLOV mixed with different sizes of Ag nanoparticles followed a double exponential decay trend. Once τ_1_ was fixed at 4.8 ns, the photoluminescence decay time of iLOV protein adsorbed onto 67 nm, 29 nm, and 15 nm Ag nanoparticles (τ_2_) was found to be 2.5 ns, 2.7 ns, and 2.9 ns, respectively. These results showed that the fluorescence lifetime of iLOV–Ag nanoparticles is size-dependent. The short and long lifetime components were 70.78% (2.5 ns) and 29.22% (4.8 ns) for the iLOV protein solution in the presence of 67 nm Ag nanoparticles. In the presence of 29 nm Ag nanoparticles, the short and the long lifetime components were 62.03% and 37.97%, respectively; and in the presence of 15 nm Ag nanoparticles, the short and the long lifetime components were 48.04% and 51.96%, respectively. Here, the long component was attributed to unbound iLOV protein and the short component was attributed to protein adsorbed onto Ag nanoparticles. The time-resolved fluorescence lifetime of iLOV–Ag decreased as the size of the Ag nanoparticles increased.

Due to errors in excitation light intensity, fluorescence lifetime analysis is more reliable than fluorescence intensity analysis. Therefore, if the interaction between iLOV and Ag nanoparticles occurs through the SET process, then the fluorescence lifetime can be utilized to calculate the energy-transfer efficiency. The energy-transfer efficiency can be calculated using the equation below.

*ϕ*_ET_ = 1 − *τ*_2_/*τ*_1_, (1)

where *τ_1_* and *τ_2_* represent the fluorescence lifetimes of iLOV and iLOV adsorbed onto Ag nanoparticles, respectively. Assuming that the observed lifetime decrease was entirely caused by the photoinduced energy-transfer process, the energy-transfer efficiency between iLOV and differently sized Ag nanoparticles was calculated according to the above equation. The results showed that with the increase of Ag nanoparticle size, the value of τ_2_ decreased as the energy-transfer efficiency increased. This result indicated the size-dependent effect of energy-transfer efficiency. The fitting results are shown in Table 2.

Equation (2) shows the quantum efficiency of energy transfer in surface energy-transfer process. It has been proven that if SET occurs, the transfer efficiency between the donor and acceptor is distance-dependent and can be calculated using Equation (2) [10].

*ϕ*_SET_ = 1/(1 + (*d*/*d*_0_)^4^). (2)

where *d* represents the distance between the centers of the donor and recipient. *d_0_* is the distance at which a dye will display equal probabilities of energy transfer and spontaneous emission. Because the size of iLOV was fixed in our experiments, the energy-transfer efficiency was expected decrease with increasing size of the Ag nanoparticles, according to the above equation. Transfer efficiency increased with the increase of Ag particle size in our experiments. Therefore, it was concluded that the interaction between iLOV and Ag nanoparticles was not through the surface energy-transfer process but through the surface electron-transfer process.

### 2.6. Electron-Transfer Pathway between iLOV and Ag Nanoparticles

**Figure 6:**
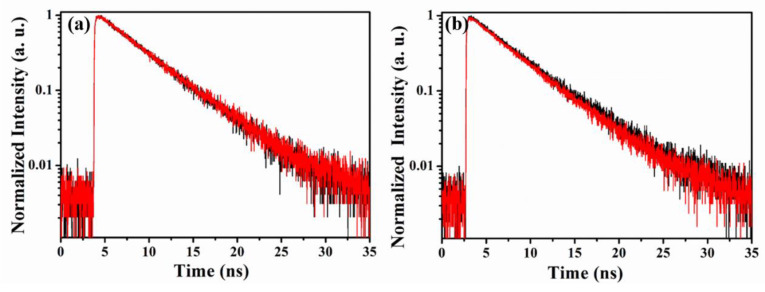
(**a**) Time-resolved fluorescence of riboflavin with (red) and without Ag nanoparticles (black). (**b**) Time-resolved fluorescence of iLOV-W83A with (red) and without Ag nanoparticles (black).

To further investigate the surface electron-transfer process between iLOV and Ag nanoparticles, the following experiments were conducted using 67 nm Ag nanoparticles. The iLOV protein molecule is composed of two major parts: the FMN fluorophore and the amino acids surrounding the FMN. Riboflavin, which has a similar structure and biologically active forms as FMN except for a phosphate group, was utilized to figure out which part was responsible for the electron-transfer process. As shown in Figure 6a, no change of the fluorescence lifetime was observed after Ag nanoparticles were added to riboflavin in buffer solution. This indicated that there was no electron transfer between riboflavin and Ag nanoparticles. According to the results above, one could speculate that the amino acids surrounding FMN mediate the electron-transfer process between iLOV and Ag nanoparticles. Because there are 20 kinds of amino acid, the active sites of electron transfer between iLOV and Ag nanoparticles remains to be determined. Among all the amino acids, tryptophan makes a good mediator for electron transfer due to its isoelectric point of 5.89. Furthermore, the short distance (10.4 Å) between tryptophan and FMN may facilitate the electron-transfer process. Therefore, tryptophan might be the active site of electron transfer between iLOV and Ag nanoparticles.

We mutated the sole tryptophan in the iLOV protein to alanine to produce iLOV-W83A, in order to further investigate the electron-transfer pathway of iLOV to Ag nanoparticles. We performed fluorescence lifetime measurements on iLOV-W83A with and without Ag nanoparticles. The time-resolved fluorescence spectra of iLOV-W83A protein with and without the presence of Ag nanoparticles are shown in Figure 6b. The results showed little difference between the fluorescence lifetimes of iLOV-W83A with and without Ag nanoparticles, demonstrating no electron transfer between iLOV-W83A and Ag nanoparticles. This finding suggested that electron transfer between iLOV and Ag nanoparticles takes place through the tryptophan residue, and also confirmed that this tryptophan residue acts as the electron donor that transfers an electron to the cofactor FMN in the photoreduction of the LOV protein.

Although we preliminarily excluded the FRET process between iLOV and Ag nanoparticles according to the data involving the interaction of iLOV with 29 nm Ag nanoparticles in Section 2.4. It cannot be neglected, however, that there is a small overlap of the emission spectrum of iLOV protein and the absorbance spectrum of Ag nanoparticles, especially for the 67 nm Ag nanoparticles in the range of 480 to 520 nm. Here, the unchanged fluorescence lifetime of cofactor FMN in both iLOV and iLOV-W83A with the presence of 67 nm Ag nanoparticles served as a key result to exclude FRET.

Through a series of experiments, we finally demonstrated that the interaction between iLOV and Ag nanoparticles is realized through electron transfer between the sole tryptophan in the iLOV structure and Ag nanoparticles. In addition, we also found that the electron transfer between iLOV and Ag nanoparticles was related to the size of Ag nanoparticles; when the size of Ag nanoparticles was 67 nm, 29 nm, and 15 nm, respectively, the electron-transfer efficiency decreased with respect to the decrease in Ag nanoparticle size. This may have been due to the surface plasmon resonance (SPR) characteristics of Ag nanoparticles. The SPR effect of Ag nanoparticles has been proven to enhance the Raman scattering characteristics of molecules adsorbed onto their surface, and this enhancement is related to the size of the Ag nanoparticles [36]. Perhaps the electron-transfer process was enhanced by Ag nanoparticles in our experiments, but, as discussed by Lai et al. [9], there are many factors affecting the electron-transfer rate or efficiency. Further investigation is still required to present enough evidences to discuss more details about the electron-transfer pathways or mechanisms.

## 3. Materials and Methods

### 3.1. Materials

Ammonia water, silver nitrate (AgNO_3_), sodium citrate (Na_3_C_6_H_5_O_7_·2H_2_O), hydrazine hydrate (N_2_H_4_·H_2_O), glutathione (GSH), and phenylmethanesulfonyl fluoride (PMSF) were purchased from Sigma-Aldrich (American). Isopropylthio-β-d-galactoside (IPTG), Tris-HCl (pH 8.0), thrombin protease, and GST tag were purchased from Invitrogen (American). All chemicals and reagents were used as received without further purification.

### 3.2. Methods

#### 3.2.1. Synthesis of Ag Nanoparticles and iLOV–Ag Nanoparticles

Various sizes of Ag nanoparticles were prepared according to the methods reported in the literature [37]. Briefly, a silver ammonia solution was prepared by adding ammonia water (1 M) to 20 mL of 10 mM silver nitrate and incubating the mixture in the dark until the precipitate dissolved. A sodium citrate (20 mL, 1 mM) and hydrazine hydrate (20 mL, 3 mM) solution was then stirred into the silver ammonia solution. The color of the solution changed from colorless to red or purple-red, indicating the formation of Ag nanoparticles. The size of the formed Ag nanoparticles was related to the reaction temperature: when the reaction temperatures were set as 0 °C, 35 °C, and 70 °C, 67 nm, 29 nm, and 15 nm Ag nanoparticles were formed, respectively.

iLOV–Ag was prepared by mixing the as-prepared Ag nanoparticles with an excess of iLOV in molar ratios ranging from 10^3^ to 10^6^. The concentrations of differently sized Ag nanoparticles were roughly calculated by assuming that the percent conversion of AgNO_3_ to Ag nanoparticles was 100%. The estimated concentrations of 15 nm, 29 nm, and 67 nm Ag nanoparticles were 3.87 × 10^−8^ M, 5.25 × 10^−9^ M, and 4.35 × 10^−10^ M, respectively. The concentration of iLOV protein was determined using steady-state absorption spectroscopy. Different volumes of Ag nanoparticles were added into 1 mL of 5 × 10^−6^ M iLOV protein to form iLOV–Ag nanoparticles.

#### 3.2.2. Characterization

The size distribution of Ag nanoparticles was measured and analyzed using a dynamic light scattering (DLS) system (Horiba, SZ-100) at room temperature. UV-vis absorption spectra were recorded using a Cary 5000 UV-Vis-NIR spectrophotometer. The fluorescence emission spectrum was acquired on a PerkinElmer Fluorescence Spectrometer LS55 using an excitation wavelength of 430 nm.

#### 3.2.3. Time-Resolved Fluorescence Measurements

The fluorescence lifetimes were recorded by time-correlated single-photon-counting (TCSPC) measurement using a home-built fluorescence lifetime setup (Harp300, Picoquant, Berlin, Germany). The excitation light source was a broadband tunable pulse femtosecond Ti:sapphire laser (Chameleon Ultra II, Coherent Inc., Santa Clara, CA, USA) equipped with a pulse-selection system (Model 305, Conoptics, Danbury, CT, USA) to modulate the repetition rate. A pulsed excitation laser of 450 nm was generated from the second harmonic generation with a BBO nonlinear crystal. Two long-pass filters (AT470lp, Chroma, Bellows Falls, VT, USA) were utilized to filter out the excitation light. Sample fluorescence was collected by a single-photon-counting avalanche photo diode detector (MPD, Picoquant, Berlin, Germany) and delivered into the TCSPC system for analysis. The typical full width at half-maximum (FWHM) of the system response was 0.15 ns. The recorded data were plotted as a fluorescence lifetime histogram, and the dynamic parameters were obtained from single or double exponential fitting.

#### 3.2.4. Protein Expression and Purification

iLOV and iLOV-W83A were expressed and purified from *Escherichia coli* Rosetta strains. First, 10 μL of fresh Rosetta strains were seeded in 5 mL LB-ampicillin medium with shaking, cultured at 37 °C and 220 rpm until the OD (optical density) value of the strains at 600 nm reached 0.5. Isopropylthio-β-d-galactoside (IPTG) with a final concentration of 0.1 μM was added into 10 min ice-bathed Rosetta suspension. After that, the suspension was cultured with shaking for 20 h at 16 °C and 160 rpm to induce the expression of iLOV. The cells were then collected by centrifugation for 30 min at 4500 rpm, and then resuspended in 30 mL Tris-HCl (pH 8.0) buffer solution. After adding 100 μL of 0.2 M phenylmethanesulfonyl fluoride (PMSF), the cell suspension was broken up at 4 °C and sonicated on ice for 5 min with 5 s rest intervals. The sonicated suspension was subjected to ultracentrifugation at 10,000 rpm for 1 h, and the resulting supernatant lysate was applied to a GST resin column which was then washed with at least three bed volumes of buffer solution to remove impurities. The column was treated with buffer solution and thrombin protease for 12–14 h at 16 °C with vigorous shaking at 220 rpm to cut off the GST tag and isolate the target protein, and then washed with GSH solution to remove the GST tag. The expression of iLOV protein was qualitatively analyzed via sodium dodecyl sulfate polyacrylamide gel electrophoresis (SDS-PAGE), and the concentration of the target protein was determined by UV-vis spectroscopy according to the absorption at 280 nm and 410 nm.

## 4. Conclusions

In this work, we investigated the interaction between silver nanoparticles and iLOV proteins through steady-state and time-resolved fluorescence spectroscopy methods. The optical spectroscopic study of the hybrid system obtained by the conjugation of iLOV protein to Ag nanoparticles of different sizes provided information on the interactions occurring at the interface between iLOV protein and the Ag nanoparticle surface, and on the resulting photophysical properties of the system. This phenomenon was explained by the increase in the nonradiative decay rate as a consequence of electron transfer at the iLOV and Ag interface. We demonstrated herein that photoinduced electron transfer between the iLOV protein and Ag nanoparticles occurs rapidly and in a size and concentration-dependent manner. We used riboflavin and iLOV-W83A to identify that the tryptophan-containing domain of iLOV, specifically, interacts with Ag nanoparticles. These results provide a deeper understanding of the structural features responsible for protein–nanoparticle binding and also present possibilities for better modeling and prediction of the interaction of proteins and other biomolecules to nanoparticles.

## Figures and Tables

**Table 1:** The fitting results of fluorescence decay curves of iLOV protein with various quantities of 29 nm Ag nanoparticles added.

Ag Nanoparticles	0 μL	5 μL	20 μL	140 μL
A1 (τ_1_ = 4.8 ns)	100%	63.98%	49.75%	37.97%
A2 (τ_2_ = 2.7 ns)	0	36.02%	50.25%	62.03%
Fitting equation: I(t) = I_0_ + A_1_exp(−t/τ_1_) + A_2_exp(−t/τ_2_)				

**Table 2:** Time-resolved fluorescence lifetimes and transfer efficiencies of free iLOV and iLOV adsorbed onto silver nanoparticle surfaces.

System	*τ*_1_ (ns)	*τ*_2_ (ns)	Efficiency (%)
Free iLOV	4.8		
iLOV with 67 nm Ag nanoparticles	4.8	2.5	47.92
iLOV with 29 nm Ag nanoparticles	4.8	2.7	43.75
iLOV with 15 nm Ag nanoparticles	4.8	2.9	39.58

*τ*_1_ represents the lifetime of free protein, *τ*_2_ represents the lifetime of absorbed protein.

## References

[1] Khlebtsov N.G., Dykman L.A. (2010). Optical properties and biomedical applications of plasmonic nanoparticles. J. Quant. Spectrosc. Radiat. Transf..

[2] Zhang H., Zhu Y., Qu L., Wu H., Kong H., Yang Z., Chen D., Mäkilä E., Salonen J., Santos H.A. (2018). Gold nanorods conjugated porous silicon nanoparticle encapsulated in calcium alginate nano hydrogels using microemulsion templates. Nano Lett..

[3] Garabagiu S.A. (2011). spectroscopic study on the interaction between gold nanoparticles and hemoglobin. Mater. Res. Bull..

[4] Alipilakkotte S., Sreejith L. (2018). Green synthesized PLA/silver nanoparticle probe for sensing of hydrogen peroxide in biological samples. Mater. Lett..

[5] Xie C., Zhen X., Lyu Y., Pu K. (2017). Nanoparticle Regrowth Enhances Photoacoustic Signals of Semiconducting Macromolecular Probe for In Vivo Imaging. Adv. Mater..

[6] Li Y., Schluesener H.J., Xu S. (2010). Gold nanoparticle-based biosensors. Gold Bull..

[7] Blanco E., Shen H., Ferrari M. (2015). Principles of nanoparticle design for overcoming biological barriers to drug delivery. Nat. Biotechnol..

[8] Zaimy M.A., Saffarzadeh N., Mohammadi A., Pourg hadamyari H., Izadi P., Sarli A., Moghaddam L.K., Paschepari S.R., Azizi H., Torkamandi S. (2017). New methods in the diagnosis of cancer and gene therapy of cancer based on nanoparticles. Cancer Gene Ther..

[9] Lai Y., Lin C., Chung M., Hung P., Horng J., Chen I., Chu L. (2017). Distance-dependent excited-state electron transfer from tryptophan to gold nanoparticles through polyproline helices. J. Phys. Chem. C.

[10] Sen T., Haldar K.K., Patra A. (2008). Au nanoparticle-based surface energy transfer probe for conformational changes of BSA protein. J. Phys. Chem. C.

[11] Zu F., Yan F., Bai Z., Xu J., Wang Y., Huang Y., Zhou X. (2017). The quenching of the fluorescence of carbon dots: A review on mechanisms and applications. Mikrochim. Acta.

[12] Farquhar G.D., von Caemmerer S.V., Berry J.A. (1980). A biochemical model of photosynthetic CO_2_ assimilation in leaves of C3 species. Planta.

[13] Das M.R., Sarma R.K., Saikia R., Kale V.S., Shelke M.V., Sengupta P. (2011). Synthesis of silver nanoparticles in an aqueous suspension of graphene oxide sheets and its antimicrobial activity. Colloid Surface B.

[14] Farooq S., Nunes F.D., de Araujo R.E. (2018). Optical properties of silver nanoplates and perspectives for biomedical applications. Photonic Nanostruct..

[15] La Spada L., Vegni L. (2018). Electromagnetic nanoparticles for sensing and medical diagnostic applications. Materials.

[16] McFarland A.D., Van Duyne R.P. (2003). Single silver nanoparticles as real-time optical sensors with zeptomole sensitivity. Nano Lett..

[17] Khatami M., Varma R.S., Zafarnia N., Yaghoobi H., Sarani M., Kumar V.G. (2018). Applications of green synthesized Ag, ZnO and Ag/ZnO nanoparticles for making clinical antimicrobial wound-healing bandages. Sustain. Chem. Pharm..

[18] Chandirika J.U., Annadurai G. (2018). Biosynthesis and Characterization of Silver Nanoparticles Using Leaf Extract Abutilon indicum. Glob. J. Biotechnol. Biochem..

[19] Khatami M., Norr F.G., Ahmadi S., Aflatoonia M. (2018). Biosynthesis of Ag nanoparticles using Salicornia bigelovii and its antibacterial activity. Electron. Physician..

[20] Arboleda D.M., Santillán J.M., Arce V.B., van Raap M.B.F., Muraca D., Fernández M.A., Scaffardi L.B. (2018). A simple and “green” technique to synthesize long-term stability colloidal Ag nanoparticles: Fs laser ablation in a biocompatible aqueous medium. Mater. Charact..

[21] Bondarenko O., Juganson K., Ivask A., Kasemets K., Mortimer M., Kahru A. (2013). Toxicity of Ag, CuO and ZnO nanoparticles to selected environmentally relevant test organisms and mammalian cells in vitro: A critical review. Arch. Toxicol..

[22] Bhunia A.K., Samanta P.K., Aich D., Saha S., Kamilya T. (2015). Biocompatibility study of protein capped and uncapped silver nanoparticles on human hemoglobin. J. Phys. D Appl. Phys..

[23] Delfino I., Cannistraro S. (2009). Optical investigation of the electron transfer protein azurin-gold nanoparticle system. Biophys. Chem..

[24] Kopka B., Margerl K., Savitsky A., Davari M.D., Rollen K., Bocola M., Dick B., Schwaneberg U., Jaeger K.E., Krauss U. (2017). Electron transfer pathways in a light, oxygen, voltage (LOV) protein devoid of the photoactive cysteine. Sci. Rep.-UK..

[25] Ravikumar Y., Nadarajan S.P., Lee C.S., Rhee J.K., Yun H. (2015). A new generation fluorescent based metal sensor-iLOV protein. J. Microbiol. Biotechnol..

[26] Wang S.E., Brooks A.E., Cann B., Simoes-Barbosa A. (2016). The fluorescent protein iLOV outperforms eGFP as a reporter gene in the microaerophilic protozoan Trichomonas vaginalis. Mol. Biochem. Parasit..

[27] Dang X., Chalkias S., Koralnik I.J. (2015). JC virus-iLOV fluorescent strains enable the detection of early and late viral protein expression. J. Virol. Methods.

[28] Acikgoz S., Ulusu Y., Akin S., Sonmezoglu S., Gokce I., Inci M.N. (2014). Photoinduced electron transfer mechanism between green fluorescent protein molecules and metal oxide nanoparticles. Ceram Int..

[29] Khrenova M.G., Nemukhin A.V., Domratcheva T. (2015). Theoretical characterization of the flavin-based fluorescent protein iLOV and its Q489K mutant. J. Phys. Chem. B.

[30] Avouris P., Persson B.N. (1984). Excited states at metal surfaces and their nonradiative relaxation. J. Phys. Chem..

[31] Davari M.D., Kopka B., Wingen M., Bocola M., Prepper T., Jaeger K.E., Schwaneberg U., Krauss U. (2016). Photophysics of the LOV-based fluorescent protein variant iLOV-Q489K determined by simulation and experiment. J. Phys. Chem. B.

[32] Dulkeinth E., Ringler M., Klar K.A., Feldmann J., Murioz A., Parak W.J. (2005). Gold nanoparticles quench fluorescence by phase induced radiative rate suppression. Nano Lett..

[33] Cannone F., Chirico G., Bizzarri A.R., Cannistraro S. (2006). Quenching and blinking of fluorescence of a single dye molecule bound to gold nanoparticle. J. Phys. Chem. B.

[34] Zhang J., Fu Y., Lakowicz J.R. (2007). Enhanced forster resonance energy transfer (FRET) on a single metal particle. J. Phys. Chem. C.

[35] Liu T., Zhong J., Gan X., Fan C., Li G., Matsuda N. (2003). Wiring electrons of cytochrome C with silver nanoparticles in Layered Films. Chemphyschem.

[36] Mondal S., Rana U., Malik S. (2015). Facile decoration of polyaniline fiber with Ag nanoparticles for recyclable SERS substrate. ACS Appl. Mater. Inter..

[37] Li J.J., Liu C.L., Ren X.Y., Yang H.J., Yang J.X., Yong H. (2011). Preparation of high-stable and monodisperse colloidal silver nanoparticles. Precious Metal..

